# Atrial Fibrillation in the Elderly: The Role of Sub-Clinical Isolated Cardiac Amyloidosis

**DOI:** 10.1038/s41598-019-53119-z

**Published:** 2019-11-12

**Authors:** Darshan Krishnappa, Richard Dykoski, Ilknur Can, Mackenzie Mbai, Inder S. Anand, Viorel Florea, Y. S. Chandrashekar, Jian-Ming Li, Venkatakrishna N. Tholakanahalli

**Affiliations:** 1 Cardiovascular Division, University of Minnesota, Minneapolis, Minnesota USA; 2 Cardiovascular Division, Minneapolis Veterans Affairs Health Care System, Minneapolis, Minnesota USA; 3 Department of Pathology, Minneapolis Veterans Affairs Health Care System, Minneapolis, Minnesota USA; 4 Cardiovascular Division, Veterans Affairs Medical Centre, San Diego, California USA

**Keywords:** Atrial fibrillation, Cardiomyopathies

## Abstract

Amyloid infiltration of the atrium is described in patients with valvular heart disease and is associated with an increased risk for atrial fibrillation(AF) while amyloid deposits in the ventricles is increasingly being diagnosed in patients with HFpEF. The role of amyloid deposits in patients with AF without valvular heart disease, which represents the most common form of AF globally, is undefined. In this study, we sought to assess the prevalence of sub-clinical isolated cardiac amyloidosis (ICA) at autopsy and the odds of AF in these patients. A total of 1083 patients were included in the study and 3.1% of patients were found to have asymptomatic ICA. Patients with ICA were older and had a higher odds of AF independent of age and CHA_2_DS_2_VASc score. Amongst patients with AF, those with ICA were more likely to have persistent forms of AF and had a lower sinus rhythm P-wave amplitude. Further studies are required to further define this entity, identify imaging modalities to aid in antemortem diagnosis of ICA and to establish the optimal management strategies in these patients.

## Introduction

Atrial fibrillation (AF) is the most common cardiac arrhythmia, and is associated with considerable morbidity and mortality^1^. The prevalence of AF increases with increasing age and is related to the increasing prevalence of commorbidities and structural remodelling of the atria that is believed to occur with aging^1–6^. Inceasing atrial fibrosis is known to be associated with more frequent paroxysms of AF, persistent AF and refractoriness to medical therapy^6,7^.

Recently, a considerable proportion of elderly patients with heart failure with preserved ejection fraction (HFpEF) have been found to have isolated amyloid deposits in the heart^8,9^. Further, a proportion of patients with valvular heart disease have been found to have clinically undetected amyloid deposits on atrial biopsies obtained during cardiac surgery and such deposits have been shown to be associated with an increased risk of AF^10,11^. The role of such clinically undetected atrial amyloid deposits in the aetiopathogenesis of AF occurring in the absence of valvular heart disease has not been previously evaluated.

In this study, we sought to assess the prevalence of AF in patients with clinically undetected isolated cardiac amyloidosis (ICA) detected at autopsy and identify electrocardiographic (EKG) markers of such amyloid deposits.

## Methods

### Patient population

This retrospective study included all autopsies conducted at the Minneapolis Veterans Affairs Health Care System over a 20-year period from January 1999 to October 2018. Autopsies that did not involve an evaluation of the heart were excluded as were patients with a clinical diagnosis of plasma cell dyscrasias or amyloidosis. Further, patients with multisystem amyloid infiltration at autopsy were also excluded.

### Diagnosis of cardiac amyloidosis

**Figure 1:**
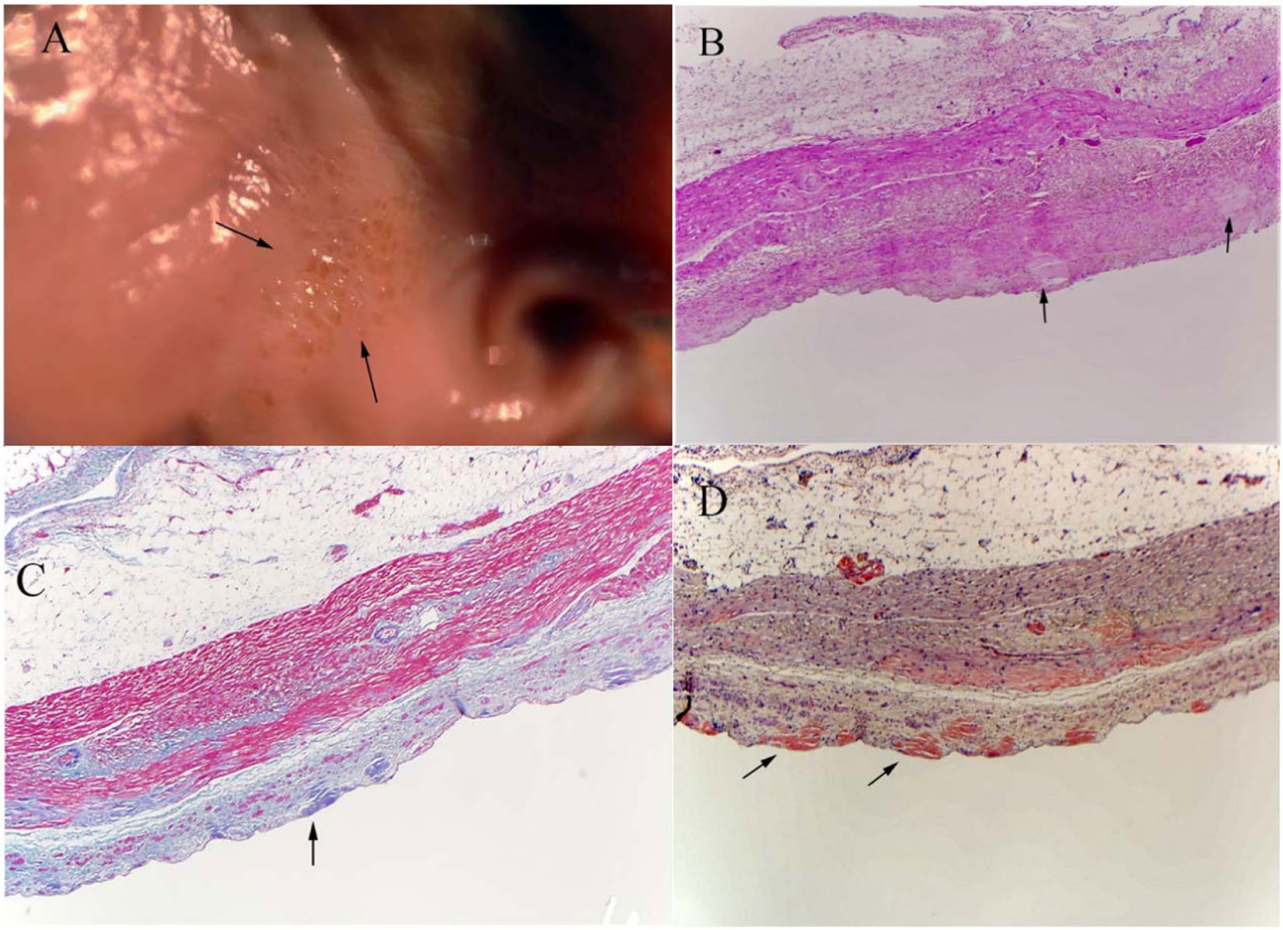
Histopathological appearance of amyloid deposits. (**A**) Gross specimen of the left atrium showing amyloid deposits (brown dots, marked by arrows) giving it the charactersitic ‘sand paper’ appearance. (**B**) Hemotoxylin and Eosin stain. (**C**) Trichrome stain and (**D**). Congo Red stain showing amyloid deposits within atrial tissue in different patients included in the study. Arrows in these images indicate amyloid deposits.

A diagnosis of amyloidosis was made using previously described criteria^12^. Briefly, the atria and ventricles were carefully examined for gross evidence of amyloid deposition. In patients with evidence of amyloid deposition, directed tissue samples were taken from affected areas. In patients without gross evidence of amyloid deposition, multiple samples were taken from different cardiac chambers. Tissue samples were also obtained from all organs and subjected to microscopic analysis. Tissue samples showing features of amyloidosis on light microscopy (Hemotoxylin and Eosin stain) were confirmed with Congo red staining (Fig. 1).

### Diagnosis of atrial fibrillation

The electronic health records (including EKGs) of all patients included in the study were reviewed to identify a diagnosis of AF and to note the presence of risk factors for AF. A CHA_2_DS_2_VASc score was calculated for all patients. LV systolic dysfunction was defined as an LV ejection fraction less than 40%. AF was categorized into paroxysmal or persistent in accordance with established definitions^13,14^. Paroxysmal AF was defined as AF that terminates spontaneously or with intervention within seven days, peristent AF was defined as AF that is sustained for more than seven days while long-standing persistent AF was defined as AF that persists continously for more than one year. The EKG of all patients with AF were also assessed during sinus rhythm for P-wave characteristics. Amongst patients with long standing persistent AF the last recorded sinus rhythm EKGs were analysed.

### Statistical analysis

The study was approved by the Minneapolis VA Health Care System Institutional Review Board(IRB) and was performed in compliance with the Declaration of Helsinki. Since this was a retrospective autopsy based study the need for informed consent from relatives was waived by the IRB. All statistical analyes were performed using SPSS version 23 (SPSS Inc. version 23.0™, IBM Corporation, Chicago, USA). First, we compared patients with ICA with those without ICA. Continuous variables were expressed as Mean ± SD. Categorical variables were described as proportions and frequencies (%). Continuous variables were compared using the Student’s t-test. Categorical variables were compared using the chi-square test or Fisher’s exact test. A binary logistic regression was performed to identify characteristics of patients with ICA including all variables with a p-value < 0.1 on univariate analysis.

A secondary analysis was performed amongst patients with AF. To identify predictors of amyloidosis, a binary logistic regression was performed. All covariates were initially assessed in a univariate fashion, and variables with a p-value < 0.1 were included in the regression analysis model.

Data will be made available upon request.

## Results

A total of 1272 autopsies were performed over the duration included in the study. Of these, seven patients had a clinical diagnosis of plasma cell dyscrasias or systemic amyloidosis, while 2 patients had evidence of multiorgan amyloid infiltration at autopsy and were excluded. Further, autopsy consisted of examination of a single organ in 180 patients who were also excluded from the study. The remaining 1083 patients were included in the analysis. Autopsies were performed when either the cause of death or the etiology of the predisposing condition was unclear. At autopsy, the most frequent cause of death included pneumonia with sepsis, acute coronary syndrome, disseminated malignancy, hepatic failure and intracranial haemorrhage.

**Figure 2:**
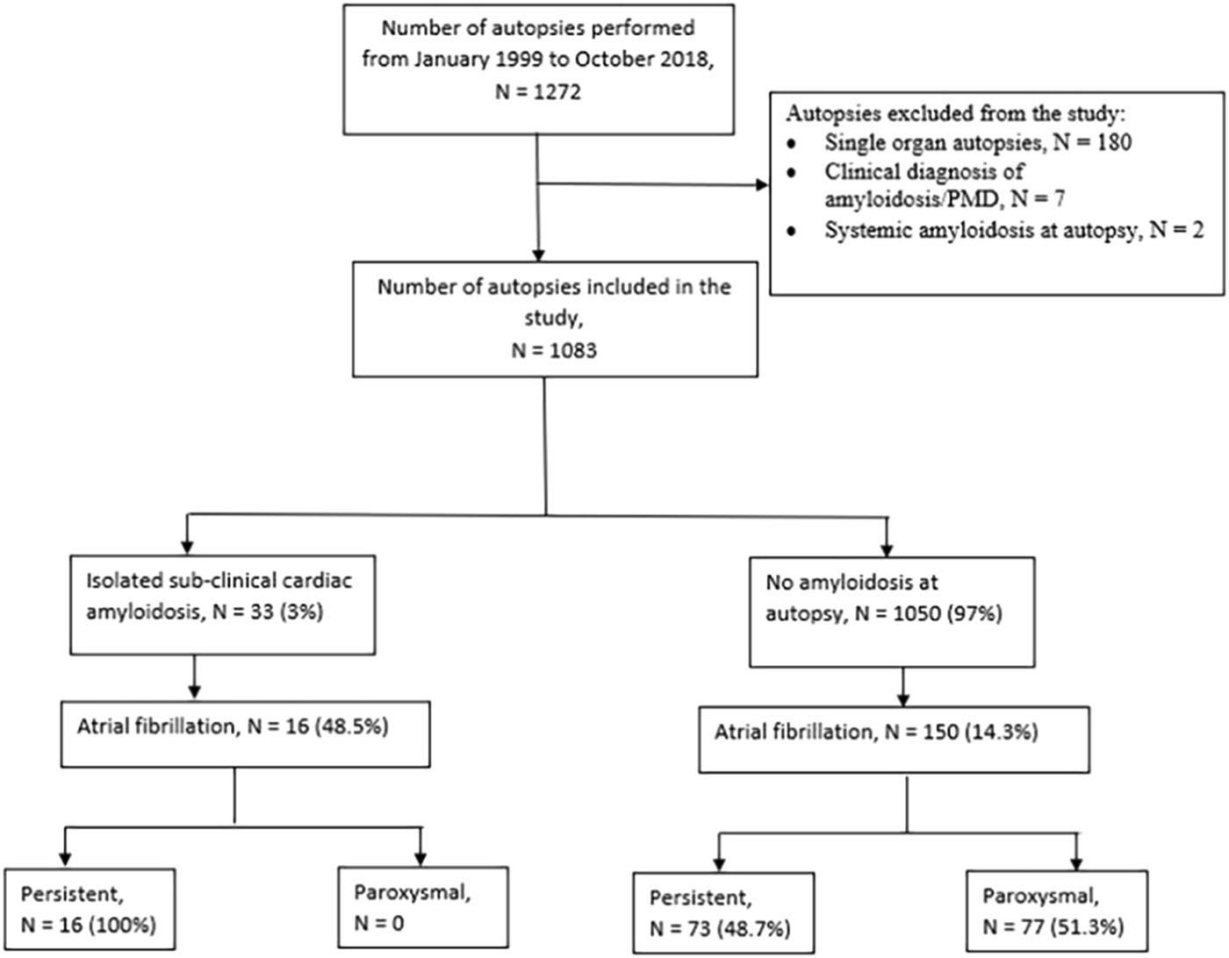
Study patients.

33 patients (3.1%) were identified to have ICA at autopsy while 1050 patients were free of amyloid deposits (Fig. 2). In patients with amyloidosis, amyloid deposits were seen both in the atria and ventricles and were either multifocal or diffuse in distribution.

**Figure 3:**
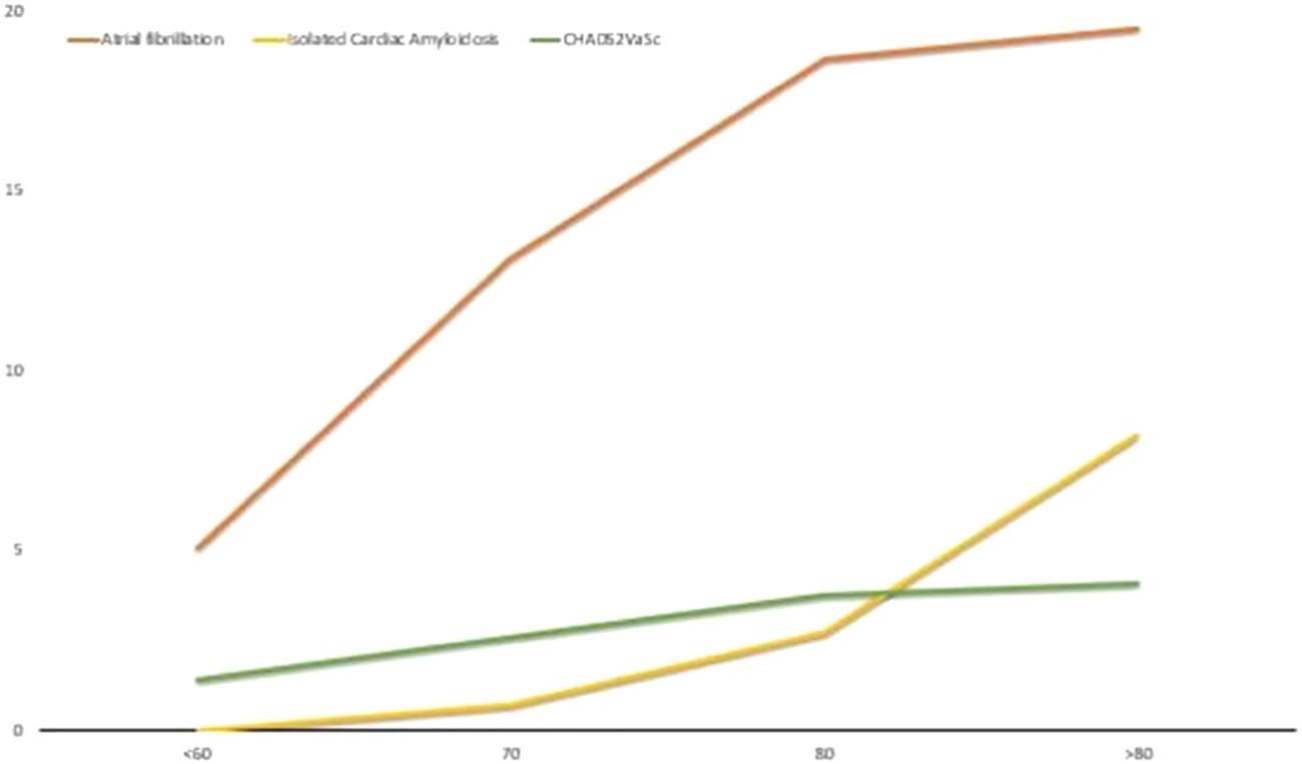
Prevalence of atrial fibrillation and Isolated Cardiac Amyloidosis at different age groups. While the prevalence of both atrial fibrillation and the CHA_2_DS_2_VASc score increase with increasing age, the increase in prevalence of atrial fibrillation is greater, likely related to other factors unaccounted for by the CHA_2_DS_2_VASc score. x-axis represents age in years; y–axis represents prevalence (%) of atrial fibrillation(red line), isolated cardiac amyloidosis (yellow line) and the CHA_2_DS_2_VASc score (green line) respectively.

The mean age of the study population was 71.4 ± 11.3 years, while the mean CHA_2_DS_2_VASc score was 3.1 ± 1.7. AF was seen in 166 (15.3%) patients. There was a gradual increase in prevalence of AF from 5.1% (9 out of 176) in individuals aged less than 60 years to 19.5% (57/292) in patients aged more than 80 years. This was associated with an increase in the mean CHA_2_DS_2_VASc score (1.4 ± 1.3 in patients aged less than 60 years to 4.1 ± 1.4 in patients aged more than 80 years) (Fig. 3). All patients with ICA were more than 60 years of age, with the prevalence increasing to 8.2% (24/292) in individuals older than 80 years. Patients with ICA were older than those without (83.9 ± 8.5 vs 71 ± 11.1 years, p < 0.001) and had a slightly higher CHA_2_DS_2_VASc score [3.8 ± 1.4 vs 3.1 ± 1.7, (mean difference −0.7) p = 0.02]. The prevalence of AF was significantly higher in these patients in comparison to patients without ICA [16 (48.5%) vs 150 (14.3%); Odds ratio (OR) 28.83, p < 0.001] with the odds of persistent AF being considerably higher [16 (48.5%) vs 73 (7%) OR 73.2, p < 0.001] in patients with ICA (Table 1).

**Table 1:** Baseline characteristics of study patients.

		No amyloidosis (N = 1050)	Amyloidosis (N = 33)	p-value
Age, in years		71 ± 11.1	83.9 ± 8.5	<0.001
Sex	Male	1045 (99.5)	33 (100)	0.7
	Female	5 (0.5)	0	
BMI, in Kg/m^2^		27.3 ± 7.1	26.6 ± 6.3	0.6
Diabetes mellitus		350 (33.3)	6 (18.2)	0.07
Hypertension		682 (65)	22 (66.7)	0.8
Stroke		176 (16.8)	8 (24.2)	0.5
Coronary artery disease		467 (44.5)	14 (42.4)	0.8
Valvular heart disease	AVR	21 (2)	1 (3)	0.9
	MVR	21 (2)	0	
	MVR + AVR	3 (0.3)	0	
LV systolic dysfunction		130 (12.4)	4 (12.1)	0.9
Atrial fibrillation		150 (14.3%)	16 (48.5%)	<0.001
CHA_2_DS_2_VASc		3.1 ± 1.7	3.8 ± 1.4	0.02

Continuous data expressed a Mean ± standard deviation; Categorical data expressed as N(%).

BMI – Body mass index, expressed in kilogram per metre square.

AVR – Aortic valve replacement.

MVR – Mitral valve replacement.

LV – Left ventricle; LV systolic dysfunction was defined as an LV ejection fraction less than 40%.

A binary logistic regression was performed to identify characteristics associated with occurrence of ICA.The regression model was statistically significant, χ^2^(4) = 71.3, *p* < 0.001. Of the four variables included in the model (age, diabetes mellitus, CHA_2_DS_2_VASc and atrial fibrillation), two characteristics, age and atrial fibrillation were found to be statistically significant. Patients with ICA tended to be older [OR −1.17; (95% CI 1.1–1.23, p < 0.001)] and had a higher odds of having atrial fibrillation [OR −5.69; (95% CI 2.58–12.55, p < 0.001)] (Table 2).

**Table 2:** Binominal logistic regression analysing charactersitics of patients with and without isolated cardiac amyloidosis.

	Odds ratio	95% confidence interval	p-value
Age	1.2	1.1–1.2	<0.001
Diabetes mellitus	0.8	0.3–2.1	0.57
CHA_2_DS_2_VASc	0.8	0.6–1.1	0.15
Atrial fibrillation	5.7	2.6–12.56	<0.001

A univariate analysis was first performed (Table 1). All variables with a p < 0.1 were included in the logistic regression model.

Amongst patients with AF, ICA was seen in 9.6% of patients, a prevalence which increased to 20.4% (10 out of 49) in patients over 80 years of age. The prevalence of ICA was also higher amongst patients with persistent AF [18% (16 of 89 patients)] while no patients with paroxysmal AF had ICA. Patients with ICA tended to be older (81.9 ± 8.4 vs 74.4 ± 9.1 years, p < 0.001) but had a similar distribution of risk factors and a similar CHA_2_DS_2_VASc score (4.4 ± 1.4 vs 4.1 ± 1.7; p = 0.4). (Table 3)

**Table 3:** Clinical characteristics of patients with atrial fibrillation.

		No amyloidosis (N = 150)	Amyloidosis (N = 16)	p-value
Age, in years		74.4 ± 9.1	81.9 ± 8.4	0.002
Sex	Male	150 (100)	16 (100)	
	Female	0	0	
BMI, in Kg/m^2^		28.1 ± 7	26.6 ± 5.1	0.4
Diabetes mellitus		55 (36.7)	4 (25)	0.4
Hypertension		100 (66.7)	12 (75)	0.5
Stroke		39 (26)	4 (25)	0.9
Coronary artery disease		105 (70)	10 (62.5)	0.5
Valvular heart disease	AVR	6 (4)	1 (3)	
	MVR	11 (7.3)	0	
	AVR + MVR	1 (0.7)	0	
LV systolic dysfunction		41 (27.3)	4 (25)	0.8
CHA_2_DS_2_VASc		4.1 ± 1.7	4.4 ± 1.4	0.4
Type of atrial fibrillation	Paroxysmal	77 (51.3)	0	<0.001
	Persistent	15 (10)	1 (6.2)	
	Long standing persistent	58 (38.7)	15 (93.8)	

Continuous data expressed a Mean ± standard deviation; Categorical data expressed as N (%).

BMI – Body mass index, expressed in kilogram per metre square.

AVR – Aortic valve replacement.

MVR – Mitral valve replacement.

LV – Left ventricle; LV systolic dysfunction was defined as an LV ejection fraction less than 40%.

### Electrocardiographic characteristics

Amongst patients with persistent and long standing persistent AF, the P-wave amplitude was lower amongst patients with ICA as compared to patients without ICA (0.71 ± 0.26 vs 1.38 ± 0.48 mV; p < 0.001) while the PR interval was longer (218.6 ± 63 vs 183.2 ± 39.7 msec, p −0.01). The P-wave duration tended to be longer but was not statistically significant (107.8 ± 41.6 vs 98.1 ± 23.3 msec, p −0.25) (Table 4).

**Table 4:** Sinus rhythm electrocardiographic characteristics of patients with atrial fibrillation.

	No amyloidosis (N = 53)	Amyloidosis (N = 14)	p-value
P-wave amplitude (in mm)	1.38 ± 0.48	0.71 ± 0.26	<0.001
P-wave duration (in milliseconds)	98.1 ± 23.3	107.8 ± 41.6	0.25
P-wave axis (in degrees)	47.3 ± 26.7	37.5 ± 35.7	0.3
PR interval (in milliseconds)	183.2 ± 39.7	218.6 ± 63	0.01
QRS amplitude (in mm)	9.8 ± 3	9.5 ± 3.8	0.9

All values are represented as Mean ± Standard deviation.

Of the 89 patients with persistent and long standing persistent atrial fibrillation, sinus rhythm electrocardiograms were available in only 67 patients and the EKG data of these patients are described in this table.

A binomial logistic regression was performed to determine the predictors of ICA in patients with persistent and long standing persistent AF. All continuous independent variables were found to be linearly related to the dependent variable. The logistic regression model was statistically significant, χ^2^(4) = 34.47, *p* < 0.001. Of the three predictor variables (age, P-wave amplitude and PR interval), age and P-wave amplitude were significant. Increasing age [OR −1.12, (95% CI, 1.02–1.24, p = 0.05)] was associated with an increased likelihood for the presence of ICA, while P-wave amplitude [OR −0.01, (95% CI, 0.01–0.14, p = 0.001)] tended to be smaller amongst patients with ICA.

## Discussion

Increasing age is an important risk factor for AF with the prevalence of AF increasing from around 2% in patients younger than 65 years to more than 15% in patients more than 80 years of age^2^. This age related increase in prevalence of AF has been suggested to be not only due to the increasing prevalence of commorbidites and structural heart disease, but also due to atrial remodelling, both structural and electrophysiological, that occurs with aging per se^5,15^. Accordingly, while some studies have suggested an increase in atrial fibrosis with aging, others have not shown such an association^7,16,17^. More recently amyloid deposition in the ventricles has been described with aging predisposing to HFpEF^8,9^. In this study, we identified clinically undetected atrial amyloid deposits in 3.1% autopsies with the prevalence increasing to 8.2% in patients greater than 80 years of age. Further, the presence of such atrial amyloid deposits was associated with a five fold higher odds of AF as opposed to patients without such amyloid deposits after adjusting for age.

Amyloid infiltration of the heart is most commonly seen in patients with AL amyloidosis or ATTR related amyloidosis. While conduction system abnormalities and atrial fibrillation is frequently seen in these patients they are typically seen in association with restrictive cardiomyopathy with heart failure dominating the clinical presentation^18–20^. More recently, Röcken *et al*. demonstrated amyloid deposits in the atrium in a series of patients undergoing cardiac surgery. A significant proportion of their patients had valvular abnormalities necessitating valve replacement surgery; the increased atrial pressure consequent to the valvular lesion was postulated to increase ANP secretion and promote the progression and consequences of isolated atrial amyloidosis^10^. Similar findings were seen by Leone and colleagues in patients undergoing surgery for valvular heart disease^11^. Both these groups demonstrated an increased risk of AF in patients with amyloid deposition. By contrast, in this study, amongst patients with amyloid deposits only one patient had valvular heart disease (aortic stenosis necessitating aortic valve replacement). Further, none of these patients had clinical manifestations of restrictive cardiomyopathy or HFpEF and thus, non-valvular AF was likely the sole manifestation of cardiac amyloid deposition in these patients. These amyloid deposits were only seen in individuals greater than 60 years of age with the prevalence increasing with increasing age (Fig. 3). Such clinically undetected cardiac amyloid deposits may partly be responsible for the higher prevalence of nonvalvular AF seen with aging.

Amyloid deposition in the atrium alters impulse conduction with the occurrence of conduction slowing and conduction blocks. Slowing of conduction within the atrium manifests as prolongation of P-wave duration. In our study, patients with ICA had a trend towards longer P-wave duration though this did not reach statistical significance. Such an increase in P-wave duration was shown in an earlier study by Röcken and colleagues^10^. Further, amyloid tissue by virtue of being inexcitable would lead to a reduction in voltage on the surface electrocardiogram, resulting in low voltage P-waves as was seen in our study. The presence of low voltage P-waves during sinus rhythm may be useful in identifying individuals with AF with amyloid deposits in the atrium. The QRS voltage though described to be lower with amyloid infiltration of the ventricles, was however not reduced likely reflecting the milder form of disease with less diffuse ventricular involvement^21^.

Amyloid deposition would lead to discontinuities in conduction within the atrium leading to re-entry and wave break predisposing to AF. The presence of multifocal or diffuse atrial amyloid deposits would create multiple drivers explaining the higher incidence of persistent AF seen both in our study and in prior studies of isolated atrial amyloidosis^10,11^.

Rhythm control in elderly patients is challenging. Rhythm control with antiarrhythmic drugs has not been shown to be superior to a rate control strategy and is also associated with a high rate of recurrence^22^. Catheter ablation in the elderly is associated with a higher rate of recurrence necessitating multiple procedures, while some studies have also shown a higher rate of mortality as compared to younger patients^23–25^. The findings of our study provide an explanation for these findings with amyloid deposits creating a greater substrate for perpetuation of AF. Further, the biophysics of lesion formation during radiofrequency ablation is influenced by underlying tissue characteristics with smaller lesions forming in scar tissue as opposed to healthy myocardium. The efficacy of lesion formation in the presence of amyloid deposits has not been studied and the presence of such amyloid deposits may limit the efficacy of ablation.

A limitation of our study is the absence of immunohistochemistry in patients with amyloid deposits thereby limiting precise identification of the type of amyloid fibril. Senile systemic amyloidosis (SSA), also known as wild-type ATTR amyloidosis, is characteristed by predominant cardiac manifestations and its prevalence increases with increasing age. Given the restriction of amyloid deposits to only the heart and the presence of such deposits in both the atria and ventricles in our patients, the pattern of amyloid deposits in our patients likely represents a mild form of SSA with isolated cardiac involvement. This is likely a prevalent form of amyloidosis in the elderly population contributing to HFpEF^8,9^ and nonvalvular AF. A second limitation of our study is the paucity of female patients who accounted for only 0.5% of the study population. This reflects the population treated at our Institution and the results of this study can thus be extrapolated only to the male population. A third limitation, inherent to the retrospective study design, is the failure to diagnose sub-clinical AF in both group of patients. The methodology employed to detect amyloid deposition in our patients included a gross examination of both atria followed by directed biopsies from affected areas. In patients with no gross evidence of amyloid deposition, multiple samples were obtained from both atria. It is possible that this method of amyloid detection couldve missed detecting patients with focal amyloid deposits and this represents an additional limitation of this study. Since there is no other method available for identifying asymptomatic (silent) cardiac amyloid deposition we are unable to comment of the sensitivity of the method employed.

Additionally, while this is an autopsy based study and thus bears with it the concern for selection bias, there currently is no modality to identify isolated atrial amyloidosis antemortem. This report represents the first description of the role for such amyloid deposits in the elderly with AF occurring in the absence of valvular heart disease and additional studies are required to further define this entity and aid in its antemortem clinical detection.

Despite these limitations, our study identifies the presence of clinically silent cardiac amyloid deposits in a significant proportion of elderly patients and recognises a higher prevalence of persistent AF in these patients. The causality of this association needs to be evaluated in further studies.

## Conclusion

Isolated cardiac amyloid deposition may occur as a mild form of SSA, with AF as the only clinical manifestation of this condition. This undetected form of cardiac amyloidosis appears to occur frequently in elderly patients presenting with AF. Increasing life expectancy and the increasing prevalence of AF amongst the elderly highlights the importance of further defining this entity and additional studies are required to identify imaging modalities to detect atrial amyloid deposits antemortem and to establish the optimum management strategy in these patients.
